# GOLPH2 expression may serve as diagnostic marker in seminomas

**DOI:** 10.1186/1471-2490-10-4

**Published:** 2010-02-25

**Authors:** Florian R Fritzsche, Glen Kristiansen, Marc-Oliver Riener, Manfred Dietel, Beibei Oelrich

**Affiliations:** 1 Institute of Surgical Pathology, UniversitätsSpital Zürich, Zurich, Switzerland; 2 Institute of Pathology, University Hospital Erlangen, Erlangen, Germany; 3 Institute of Pathology, Charité - Universitätsmedizin Berlin, Berlin, Germany; 4 Stanford Prevention Research Center, Stanford Medical School, Palo Alto, California, USA

## Abstract

**Background:**

GOLPH2 (Golgi phosphoprotein 2) is a novel Golgi membrane protein. Despite its unknown physiologic function, however, it has been proposed as a biomarker for hepatocellular and prostate carcinoma due to its upregulation in those cancer entities. Whether the overexpression of GOLPH2 is tumour specific or a generic parameter of malignancy and whether this finding is true for additional carcinomas has not been determined. In this study, we aimed to evaluate the expression pattern of GOLPH2 in testicular seminomas, the most common histologic subtype of testicular neoplasm.

**Methods:**

GOLPH2 protein expression was assessed by immunohistochemistry in 69 testicular seminomas and compared to the expression rates in matching normal testicular tissue and intratubular germ cell neoplasia of unclassified type (IGCNU). In addition, a subset of Leydig cell tumours was analyzed accordingly.

**Results:**

GOLPH2 was consistently overexpressed (89.9%) in seminomas. Matching non-neoplastic tissue showed weak or negative staining. The observed differences between non-neoplastic and neoplastic tissue were statistically highly significant (p < 0.001). There were no significant associations with tumour status. Interestingly, GOLPH2 was also highly expressed in the intertubular Leydig cells as well as in Leydig cell tumours.

**Conclusions:**

GOLPH2 protein is highly expressed in seminomas and in Leydig cell tumours. This study fosters the association of GOLPH2 with malignant neoplastic processes. The staining pattern is easily assessable and consistent which is a favourable property especially in clinical settings. GOLPH2 could be a novel immunohistochemical marker for the assessment of testicular neoplasms, especially against the background that in analogy to hepatocellular carcinomas complementary GOLPH2 serum levels might be helpful in detecting metastases or recurrent tumour. Therefore serum studies and analyses of GOLPH2 expression in non-seminomatous germ cell tumours are strongly warranted.

## Background

Testicular neoplasms represent the most common malignancy in young adults with rising incidence [1,2]. The two major categories are testicular germ cell tumours (GCT) and sex cord-stromal tumours. GCT is a highly heterogeneous group of neoplasms divided into seminomas and non-seminomas. The most frequent single histologic subtype of GCT is seminoma which consists of uniform tumour cells resembling gonocytes that are unable to undergo normal spermatogenesis owing to a blocked regular differentiation [3]. Among non-germ cell tumours, Leydig cell tumours (LCT) are the most common entity. Despite the generally benign behaviour of the majority of LCT, 10% are malignant and characterized by limited response to chemotherapy and irradiation and poor prognosis. In comparison to other GCT, seminomas have a more favourable prognosis even in advanced stages. Surgery and systemic treatment regimens in combination with radio- and chemotherapy are able to cure this malignancy in the majority of cases. Consequently, rising numbers of long term survivors have turned the focus more and more to the long term effects of chemotherapeutic treatments. Delayed toxicity and the development of secondary malignancies are a problem of many of today's anti-neoplastic armamentarium. Unfortunately, the young age of the typical seminoma patient further enhances the time at risk for the side effects of these therapies. Despite the progress in treatment GCT still remain to be a deadly disease for a small group of patients attributed to relapse and therapy resistance. Therefore, the demand remains to further characterize this tumour entity to better understand and predict the biological behaviour and to identify targets for more sophisticated and risk-adapted therapeutic options. The novel Golgi phosphoprotein 2 (GOLPH2), also known as Golgi protein 73 (GP73) and Golgi membrane protein 1 (GOLM1), has recently been described as a diagnostic marker in prostate cancer with similar characteristics as alpha-methylacyl CoA racemase [4]. Likewise, GOLPH2 mRNA could be used in a combination of markers to detect prostate cancer from urine samples [5]. Furthermore, in liver diseases several studies demonstrated the utility of GOLPH2 as a serum marker of hepatocellular carcinoma [6-10]. Since only a few studies on GOLPH2 exist until present, it is unknown whether the described upregulation and detectability in tumour tissue and various body fluids is specific for certain types of cancer. GOLPH2 could also either be generally involved in carcinogenesis similar to p53 or, more likely, given the lack of information on mutations of GOLPH2 in cancer, could be secondarily upregulated in carcinomas without any causative relations to carcinogenesis.

GOLPH2 protein is coded by the *GOLM1 *gene on chromosome 9q21.33 and was first described in giant-cell hepatitis [8]. Structurally, GOLPH2 protein consists of a short cytoplasmic N-terminal domain, a membrane-spanning region, some coiled-coil domains and a longer luminal C-terminal domain. Several possible glycosylation sites are integrated in the protein structure, but their role is still undetermined. Apart from its association with the Golgi apparatus, the functional properties of GOLPH2 are still elusive. The Golgi apparatus is a structurally highly complex central processing station which ensures proper glycosylation of secretory proteins before transported to their final destination. The critical physiologic importance of the function of the Golgi apparatus is reflected in the broad spectrum of disorders like the family of congenital disorders of glycosylation and muscular dystrophies. Modification in glycosylation has been linked to cancer metastasis. Due to the diverse and heterogeneous function of proteins trafficking through the Golgi system the location of GOLPH2 within the Golgi membrane suggests possible roles in intracellular transportation, cell signalling, protein construction and modification or structural or maintenance tasks. A recent study using a mouse model with c-terminally truncated GOLPH2 lead to decreased survival especially in females and severely impaired renal and liver epithelial organization and underscores the functional importance of GOLPH2 [11]. In this study, we analyzed the expression pattern of the novel cancer marker GOLPH2 in testicular seminomas and Leydig cell tumours.

## Methods

### Patients

Sixty-nine patients diagnosed with testicular seminoma at the Institute of Pathology, Charité - Universitätsmedizin Berlin between 1997 and 2007 were enrolled in this study. All tumours were pure seminomas without any other germ cell tumour component. Cases were selected according to tissue availability and were not stratified for any known preoperative or pathological prognostic factor. Histological diagnosis was established according to the guidelines of the World Health Organization. The study has been approved by the Charité University Ethics Committee under the title "Retrospective analysis of tissue samples by immunohistochemistry and molecular biological methods" (EA1/06/2004) on 20^**th **^September 2004.

Patient age ranged between 25 and 70 years with a median of 37. The pT status was as follows: pT1 - 50 (72.5%), pT2 - 16 (23.2%) and pT3 - 3 (4.3%). Matching normal tissue was present for 55 cases.

### Tissue Micro Array construction

A tissue-micro-array (TMA) was constructed from formalin-fixed paraffin embedded tissue as previously described [12]. We used a tissue arrayer from Beecher Instruments (Woodland, CA, USA). The punch diameter was 1.5 mm. Each case was represented by two tumour cores and according to tissue availability by two tissue cores from non-tumourous testicular parenchyma. In 12 cases intratubular germ cell neoplasia of unclassified type (IGCNU) was available.

### Immunohistochemistry

The TMA blocks were freshly cut (3 μm) and mounted on superfrost slides (Menzel Gläser, Braunschweig, Germany). Immunohistochemistry was conducted with the Ventana Benchmark automated staining system (Ventana Medical Systems, Tucson, AZ) using Ventana reagents for the entire procedure. To detect GOLPH2, a commercially available antibody (mouse monoclonal, clone 5B10, Abnova Corporation, Taipei, Taiwan, catalog number H00051280-M06, dilution 1:500) was diluted in a Ventana diluent. For primary antibody detection we used the UltraVIEW™ DAB detection kit using the benchmarks CC1m heat induced epitope retrieval. Slides were counterstained with hematoxylin, dehydrated and mounted.

### Evaluation of the immunohistochemical stainings

The immunostainings were evaluated by two genitourinary pathologists blinded for patient outcome on a multi-headed microscope. The staining intensity was evaluated with a four-tiered grading system (0 = negative, 1 = weak, 2 = moderate and 3 = strong staining intensity). We used a 10% threshold to determine positivity. To delineate between low and high levels of GOLPH2 expression we used the median staining intensity as cut-off point.

### Statistical analysis

Statistical analysis was performed using SPSS (version 16.0; SPSS Inc., Chicago, IL, USA). The statistical significance of the associations between GOLPH2 expression and clinico-pathological parameters were assessed with Fisher's exact test and χ^2^-tests. The Wilcoxon rank-sum test was used to determine intergroup differences. P values <0.05 were considered statistically significant.

## Results

**Figure 1:**
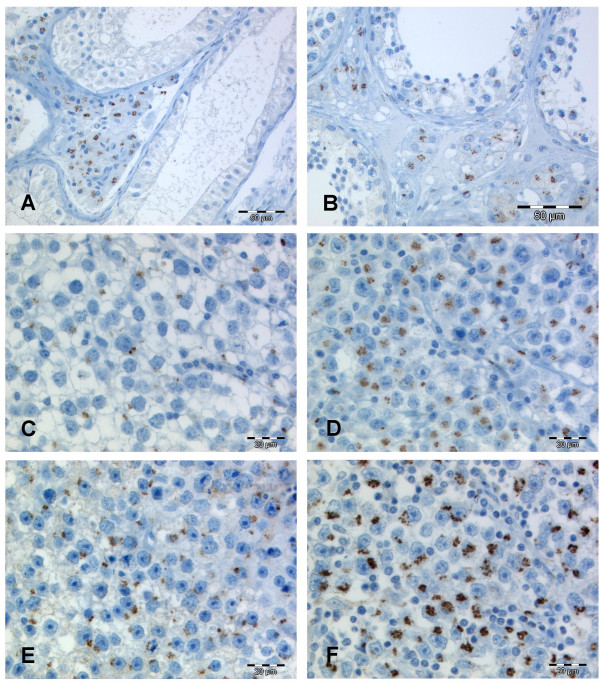
**GOLPH2 immunohistochemistry in testicular parenchyma and seminoma**. In normal testicular tubules GOLPH2 is rather absent (**A**) or faintly expressed (**B**) in the germ cell lining of the tubules. Meanwhile the intertubular Leydig cells display a pronounced perinuclear GOLPH2 staining. In seminomas we observed weak (**C**), moderate (**D/E**) and strong (**F**) granular and perinuclear GOLPH2 stainings. The majority of the seminomas displayed the moderate intensity (**D/E**) expression pattern.

**Figure 2:**
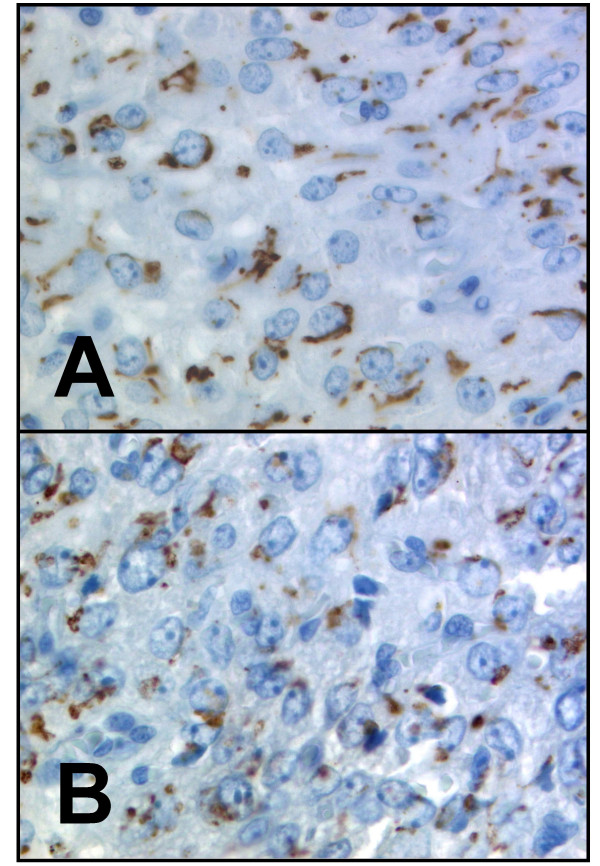
**GOLPH2 immunohistochemistry in Leydig cell tumours**. GOLPH2 expression in two exemplary Leydig cell tumours (**A/B**).

Typically GOLPH2 expression was located in the perinuclear cytoplasm, being in line with the location of the Golgi apparatus. The staining pattern was fine granular with occasional cluster formation in the seminomas (Figure 1). For 55 cases normal tissue was available. The majority of germ cells in non-neoplastic tubules were either negative or displayed a patchy slightly positive GOLPH2 staining. Most interestingly, intertubular Leydig cell clusters showed a pronounced granular GOLPH2 expression which clearly demarcated these cells from other interstitial cells and from the intratubular cells. This positivity for GOLPH2 in the Leydig cells initiated a subsequently performed immunohistological analysis of four archival Leydig cell tumours. These stainings revealed a consistent GOLPH2 expression in all four cases (Figure 2).

From the sixty-nine seminomas 54 (78.3%) displayed a moderate and 8 (11.6%) a strong GOLPH2 expression. The typical staining pattern, as described above, was consistent with that previously described in other tumours and was clearly detectable in most of the cells of the respective seminoma. Only 7 (10.1%) cases had a weak GOLPH2 staining resembling that of the normal tubular germ cells.

The observed differences between non-neoplastic and neoplastic tissue were statistically highly significant (p < 0.001). The staining of the intratubular germ cell neoplasia of unclassified type was negative (3 cases) or weak (9 cases). The GOLPH2 expression in seminomas was not associated with tumour status or patient age (Table 1).

**Table 1:** Associations (χ^2^-tests) between the protein expression GOLPH2 in seminomas and clinico-pathological parameters

	Total	GOLPH2 low	GOLPH2 high	p-value
**All cases**	69 (100%)	7 (10.1%)	62 (89.9%)	
**Age**				0.632
≤36	29 (42%)	3 (10.3%)	26 (89.7%)	
>36	40 (58%)	4 (10%)	36 (90%)	
**pT-status**				0.868
pT1	50 (72.5%)	6 (12.0%)	44 (88.0%)	
pT2	16 (23.2%)	0 (0)	16 (100%)	
pT3	3 (4.3%)	1 (33.3%)	2 (66.7%)	

## Discussion

This study demonstrates for the first time that GOLPH2 is a significantly overexpressed protein in seminomas in comparison to normal testicular parenchyma. Wright et al. previously described the location of GOLPH2 in germ cells and Sertoli cells in mouse [11]. Using the c-terminally truncated form of GOLPH2 the authors did not detect histologic morphology changes in the testis and concluded that no impairment of testicular development was involved. The overexpression in seminomas as opposed to normal germ cells reported here suggests that GOLPH2 is linked to testicular carcinogenesis. More precisely, the expression level increased from precursor lesion in IGCNU to seminomas. Our findings in testicular neoplasms mirror the previous results of our group and others in prostate cancer and hepatocellular carcinoma [4,13-15].

In addition, GOLPH2 is distinctively expressed in Leydig cells. No published data was available to date reporting on this prominent feature. Taken into consideration that GOLPH2 expression in normal tissues of multiple organs was widely found to be minimal or absent [11], this interesting finding raised the question whether the overexpression could also be detected in LCT which could be confirmed by our subsequent analysis. Current diagnosis of malignant Leydig cell tumours harbouring metastatic potential is mainly based on morphologic characteristics of tumours cells due to lack of appropriate biomarkers. Immunohistochemical markers such as Calretinin, alpha-Inhibin, S100 protein and Melan-A were shown be of some value but still lack sufficient specificity [16]. Notably, when evaluating patients with metastasis to the testis the differentiation from adrenocortical cells and melanoma cells may be problematic due to the overlapping positivity for the latter two markers [17]. Further expression analyses of GOLPH2 are warranted in particular in conjunction with clinical follow-up data to evaluate the prognostic utility of GOLPH2 as a biomarker in LCT. Given the rarity of LCT, however, studies including a larger number of cases can be challenging. Our findings indicate that GOLPH2 can be applied as a marker to identify Leydig cells and Leydig cell tumours. For differential diagnostic considerations in comparison to other sex cord-stromal tumours further investigations are needed.

It seems likely that GOLPH2 upregulation is a characteristic finding of malignant tissues not only restricted to the few cancer types that were previously described. Our research group was able to confirm the expression of GOLPH2 in hepatocellular carcinoma [14], but we also detected GOLPH2 in various other malignancies on a multi-tissue-micro-array which argues against a hepato- or prostate-specificity of this biomarker [18]. Interestingly, there is a circulating form of GOLPH2 found in hepatocellular carcinoma [6]. Serum levels of GOLPH2 were higher in cancer patients when compared to healthy individuals, which lead to the proposal of GOLPH2 as a novel serum marker in this entity [10,19]. Here, we did not include an analysis of blood samples. Studies addressing the question whether GOLPH2 serum levels of seminoma patients differ from those of healthy individuals and whether serum GOLPH2 could be used as a clinical marker to detect early disease relapse may be of particular clinical interest.

We did not find associations with tumour status or patient age. Considered that the expression pattern was consistently high among all samples, this was not surprising. Comparably, no prognostically relevant associations between GOLPH2 and the above mentioned parameters was found in our previous studies on prostate cancer, hepatocellular carcinoma or renal cell cancer [4,14,18].

The marked GOLPH2 expression in normal Leydig cells and Leydig cell tumours suggest that GOLPH2 may have more specific physiologic functions in those cells. GOLPH2 upregulation in a liver cell line by adenovirus infection further supports the view that apart from neoplastic processes other causes exist for the upregulation and expression of GOLPH2 such as inflammation and increased endocrine function [9,20]. Leydig cells are endocrine active cells secreting a variety of hormones including androgens and estrogens. In the prostate, GOLPH2 is also strongly upregulated in benign hyperplastic glands of the transitional zone. Both androgenic and estrogenic stimulation are causally related to prostatic hyperplasia [21]. In fact, normal function of Leydig cells in the testes are essential for the development of this condition as hyperplasia doesn't occur in men castrated before onset of puberty. Auxillary studies will provide more insight to the functional properties of GOLPH2 in endocrine tissue, inflammatory processes and malignant tumours.

## Conclusion

GOLPH2 protein is significantly upregulated in seminomas of the testis in comparison with non-neoplastic testicular germ cells. The staining pattern is easily assessable and consistent which is a highly favourable property especially in clinical settings. No associations with clinico-pathological characteristics were observed. Although the overexpression in seminomas and Leydig cell tumours foster the association of GOLPH2 with malignant neoplastic processes, the high expression rates in normal Leydig cells may suggest a more differentiated picture and underlines the need for functional analyses of GOLPH2 in the different physiologic and pathologic circumstances. GOLPH2 might be a novel immunohistochemical marker for seminomas, but to clarify its differential diagnostic value additional studies are warranted.

## Competing interests

The authors declare that they have no competing interests.

## Authors' contributions

FRF conceived and coordinated the study, performed immunohistological and statistical analyses and wrote the paper. GK contributed to statistical analyses and revised the paper. MOR and MD provided samples and clinico-pathological data and supported statistical analyses. BO conceived and coordinated the study and revised the paper. All authors read and approved the final manuscript.

## Pre-publication history

The pre-publication history for this paper can be accessed here:

http://www.biomedcentral.com/1471-2490/10/4/prepub
